# Supplementary figures and images for: BifA Triggers Phosphorylation of Ezrin to Benefit Streptococcus equi subsp. zooepidemicus Survival from Neutrophils Killing
Source: Biomedicines. 2022 Apr 19;10(5):932. doi: 10.3390/biomedicines10050932 (PMC9138245; doi:10.3390/biomedicines10050932)

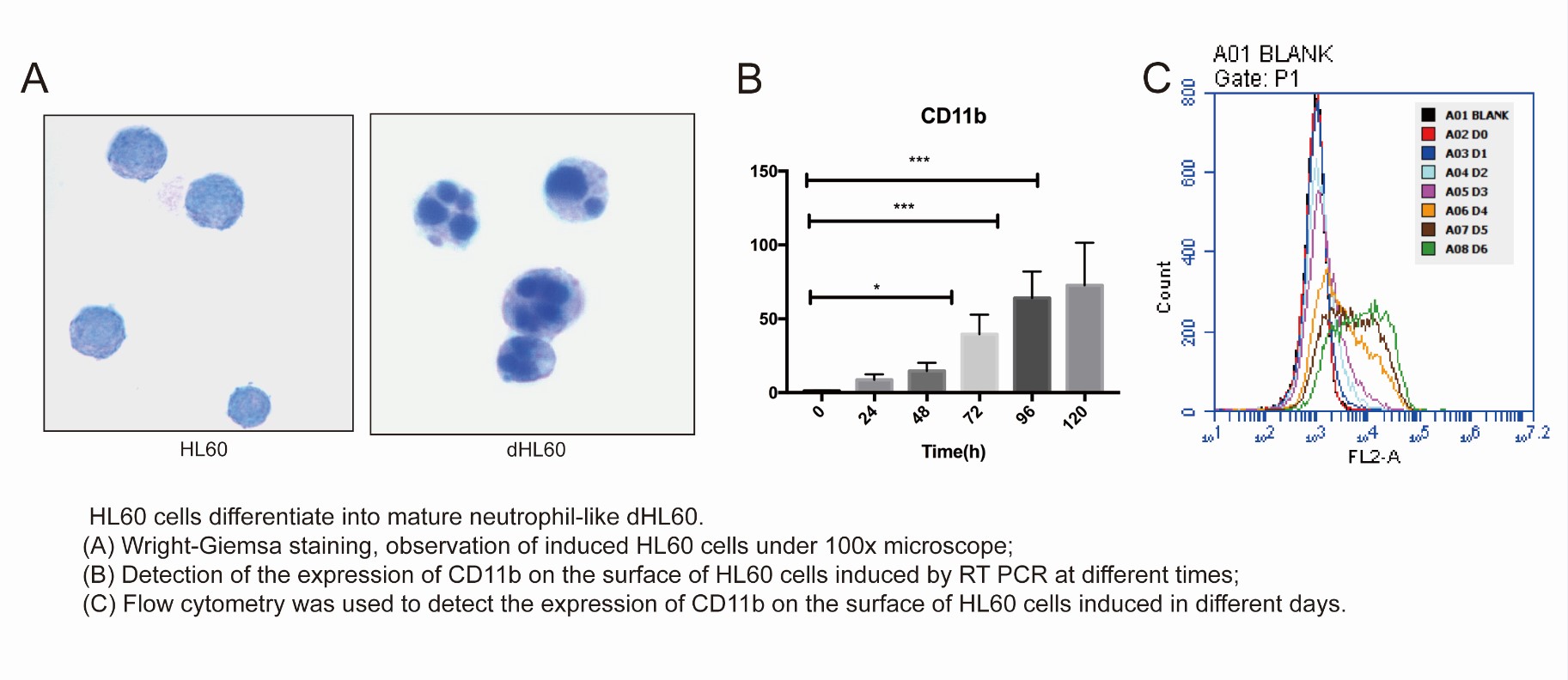

Supplement: Supplementary file 1 [file biomedicines-10-00932-s001.zip › supplementary.jpg]
